# The complete chloroplast genome sequence of *Hydrocotyle vulgaris* L. (Araliaceae)

**DOI:** 10.1080/23802359.2024.2349333

**Published:** 2024-05-17

**Authors:** Xingwu Luo, Wei Fu, Lin Li, Zhanghui Qin, Haiying Wan, Zhexian Zhang, Qiaohui Zhang

**Affiliations:** a College of Biological and Food Engineering, Hubei University for Nationalities, Enshi, China; b Enshi Tujia and Miao Autonomous Prefecture Academy of Agricultural Sciences, Enshi, China

**Keywords:** *Hydrocotyle vulgaris*, complete chloroplast genome, phylogenetic analysis

## Abstract

*Hydrocotyle vulgaris* is a perennial wetland clonal plant in the Araliaceae family, which was introduced to China as an ornamental plant in the 1990s. Although *H. vulgaris* is now considered a potential invasiveness species in China, it also plays a significant role in the remediation of water pollution. Here, we reported its complete chloroplast genome and analyzed the basic characteristics. The chloroplast genome was 153,165 bp in length, including a pair of inverted repeat (IR) regions of 25,072 bp separated by a large single-copy (LSC) region of 84,291 bp and a small single-copy (SSC) region of 18,730 bp. The *H. vulgaris* chloroplast genome contained 132 predicted genes, and its overall GC content was 37.60%. Phylogenetic analysis revealed that *H. vulgaris* was closely related to *H. verticillata*. The *H. vulgaris* chloroplast genome presented in this study will lay a foundation for further genetic and genomic studies of the genus *Hydrocotyle*.

## 1. Introduction

**Figure 1.**
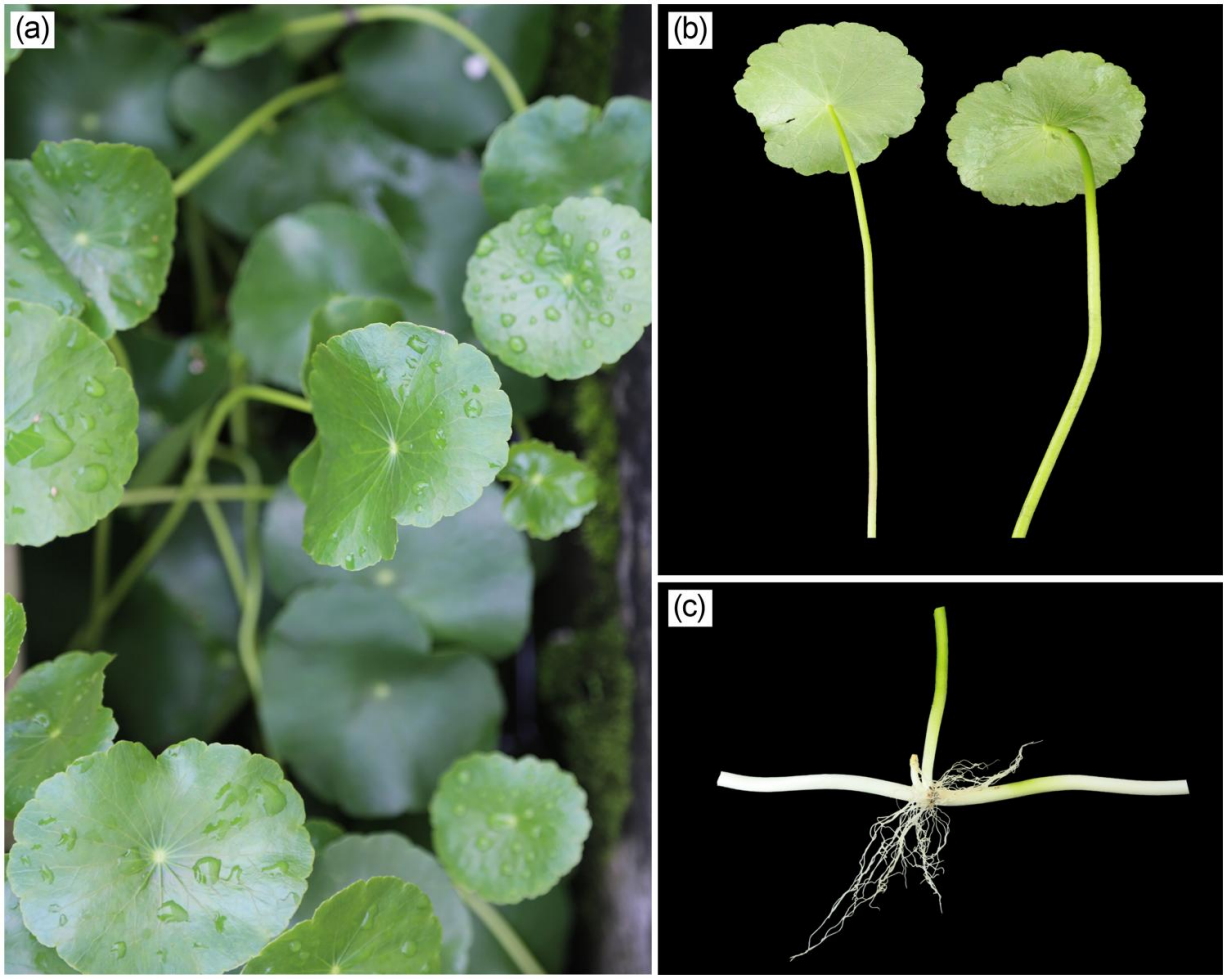
Species reference image of *H. vulgaris* (species images were taken by Dr. Lin Li at the germplasm resource nursery of Enshi Tujia and Miao Autonomous Prefecture Academy of Agricultural Sciences, Hubei Province, 2023). (a) The front side of the *H. vulgaris* leaves; (b) the abaxial side of the *H. vulgaris* leaves; (c) *H. vulgaris*’s creeping stem and the node with adventitious roots and petiolate leaves.

*Hydrocotyle vulgaris* L. 1753, belonging to the Araliaceae family, is a perennial wetland clonal plant with high morphological plasticity, rapid reproduction, and strong adaptability (Wooten 1988; Dong et al. 2015). Usually, it can form creeping stems in each of the *H. vulgaris* nodes, and each node can produce adventitious roots and petiolate leaves (Figure 1) (Dong et al. 2015; Si et al. 2020). *Hydrocotyle vulgaris* is native to Europe, America, and Africa with high adapts to different water depth changes, inhabiting moist habitats such as rivers, ponds, valleys, and dune grasslands (Jin et al. 2021; Wang et al. 2022). In the 1990s, *H. vulgaris* was introduced to China as an ornamental plant, but now it occurs in many natural and semi-natural wetlands and grasslands, such as wetlands of regions south of the Yangtze River (Liu et al. 2014; Jin et al. 2021). Nowadays, *H. vulgaris* is considered to be of potential invasiveness for its strong adaptability in both aquatic and terrestrial habitats, rapid clonal reproduction, and competitive exclusion of native species (Miao et al. 2011; Liu et al. 2014; Dong et al. 2015).

As we all know, wetland is an important ecosystem with many environmental functions on the earth (Aguilera et al. 2010; Wang et al. 2020). With human activities, some heavy metals, including cadmium (Cd), chromium (Cr), lead (Pb), and mercury (Hg), are discharged into wetlands, which severely influences the growth of wetland plants (Halpern et al. 2008; Mishra et al. 2008; Tchounwou et al. 2012). Moreover, the discharge of large amounts of nutrients such as nitrogen (N), phosphorus (P), and potassium (K) into wetlands along with wastewater can lead to eutrophication (Carpenter et al. 2006). Numerous experimental evidence indicates that *H. vulgaris* can be used in water purification to remove heavy metals and organic pollutants in wetlands (Morand et al. 2011; Ni et al. 2018; Li et al. 2020). Despite *H. vulgaris* playing an important role in the remediation of water pollution, research on its genetic diversity is very limited. Additionally, partial plastid genomic information for this species is available (Kang et al. 2023). However, characterizing the complete chloroplast genome as a reference is necessary.

In this study, we reported the first complete chloroplast genome of *H. vulgaris* and further explored the phylogenetic relationship among the *Hydrocotyle* genus, which will provide a deeper understanding of its genomic characterization and evolutionary history.

## 2. Materials

Samples of *H. vulgaris* were collected from the germplasm resource nursery of Enshi Tujia and Miao Autonomous Prefecture Academy of Agricultural Sciences, Hubei Province, China (N 30°31′53″, E 109°48′11″). A specimen was deposited at the Herbarium of Enshi Tujia and Miao Autonomous Prefecture Academy of Agricultural Sciences (Contact: Wei Fu, fuwei5@mail2.sysu.edu.cn) under the voucher number *TQC-20230901*.

## 3. Methods

Total genomic DNA was extracted from the fresh leaves using the modified cetyl trimethyl ammonium bromide (CTAB) method (Doyle and Doyle 1987). Subsequently, the DNA library was constructed by PCR reaction, and library quality was assessed on the Agilent 5400 system. Paired-end sequencing was performed on the DNBSEQ-T7 platform (Beijing Biomics Tech Co., Ltd., Beijing, China), resulting in approximately 2.89 GB of raw data. After removing the low-quality sequences and adapter sequences by fastp (Chen et al. 2018), a total of 38,541,434 clean reads were obtained to *de novo* assemble the chloroplast genome using GetOrganelle (version 1.7.1) (Jin et al. 2020) with the k-mer length: 21, 35, 45, 65, 85, and 105. With the published chloroplast genome sequences of *H. himalaica* (GenBank accession number: NC082582) as the reference, the complete chloroplast genome was annotated by CPGAVAS2 (http://47.96.249.172:16019/analyzer/annotate) (Shi et al. 2019). We further used CPGView (http://47.96.249.172:16085/cpgview/view) to improve annotation, visualize the structure of the chloroplast genome, and identify gene structures, including *cis*- and *trans*-splicing (Liu et al. 2023).

To investigate the phylogenetic relationship of *H. vulgaris*, a maximum-likelihood (ML) phylogenetic tree was constructed by IQtree (Version 1.7) (Nguyen et al. 2015) based on 18 chloroplast genome sequences downloaded from the GenBank database. The coding sequences of 78 common protein-coding genes (PCGs) were extracted from the genome annotation files using PhyloSuite (Version 1.2.2) (Zhang et al. 2020), and then multiple sequence alignment was performed by the MAFFT (Version 7.407) (Katoh and Standley 2013). The aligned sequences were end-to-end concatenated to form a supergene of each species by PhyloSuite (Version 1.2.2) (Zhang et al. 2020), and Modeltest (Version 3.7) (Posada and Crandall 1998) was used to estimate the best-fit model of nucleotide substitution.

## 4. Results

**Figure 2.**
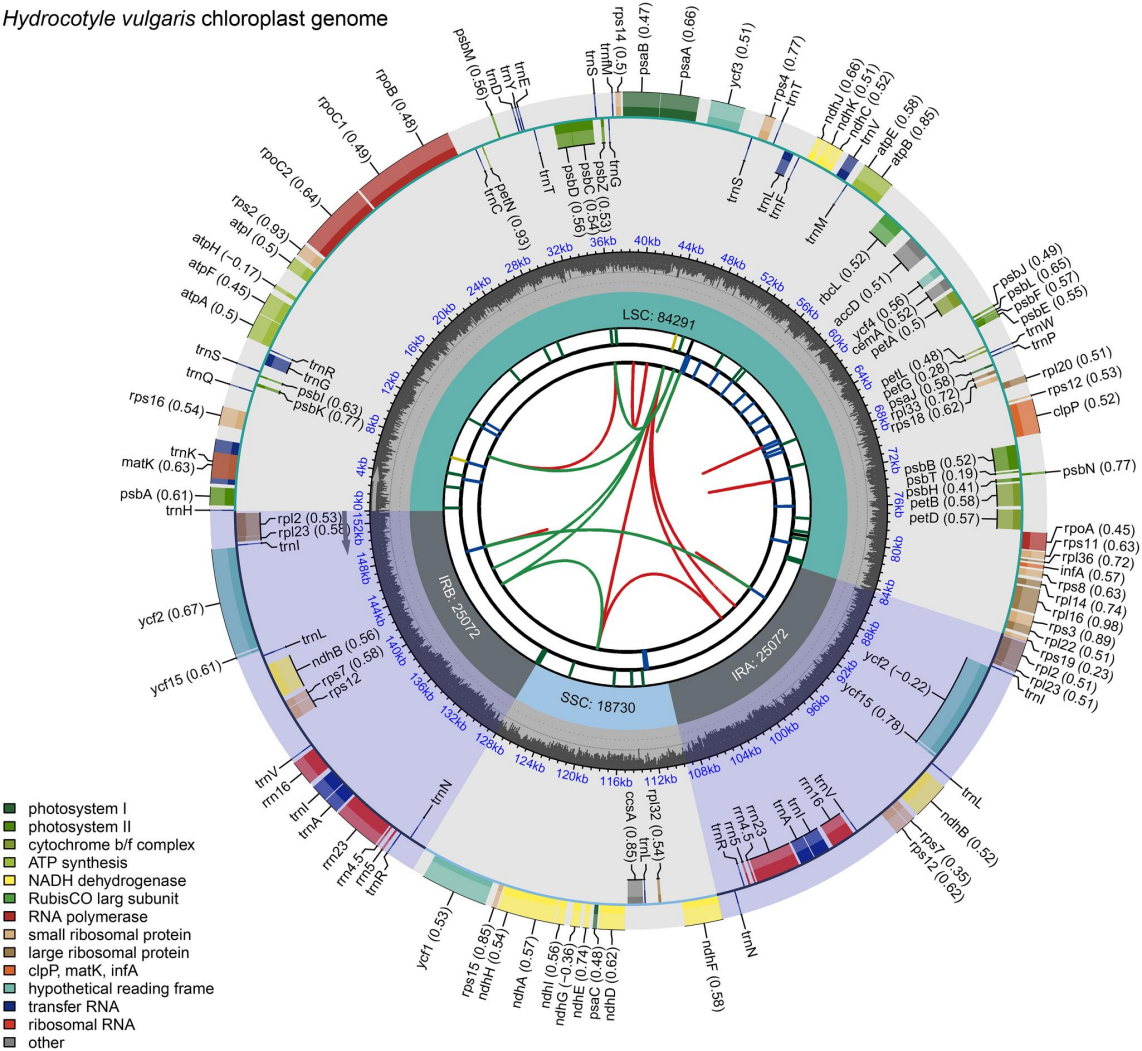
Genetic map of *Hydrocotyle vulgaris* chloroplast genome. The large single-copy (LSC) region, small single-copy (SSC) region, and two inverted repeat regions (IRA and IRB) are shown in the inside track. Gene models, including protein coding, tRNA, and rRNA genes, are shown with various colored boxes in the outer track.

The complete chloroplast genome of *H. vulgaris* (GenBank accession number: OR823205) was 153,165 bp in length with a quadripartite structure typical of angiosperms composed, including a large single-copy (LSC) region of 84,291 bp, a small single-copy (SSC) region of 18,730 bp, and inverted repeat (IR) regions of 25,072 bp (Figure 2). The overall GC content of the genome was 37.60%, with the corresponding values of 35.67%, 31.10%, and 43.27% for the LSC, SSC, and IR, respectively. The average coverage depth of the *H. vulgaris* chloroplast genome is shown in Supplementary Figure 1. This chloroplast genome encoded 132 genes, including 87 PCGs, 37 transfer RNA genes (tRNAs), and eight ribosomal RNA genes (rRNAs). Among them, 11 PCGs and nine tRNA genes had a single intron, and two PCGs (*ycf3* and *clpP*) had two introns (Supplementary Table 1). Additionally, the chloroplast genome contained 13 cis-splicing genes and the trans-splicing gene *rps12* (Supplementary Figure 2). Meanwhile, we also identified 33 simple sequence repeats (SSRs) in the chloroplast genome, including 29 mononucleotides and four dinucleotides (Supplementary Table 2).

**Figure 3.**
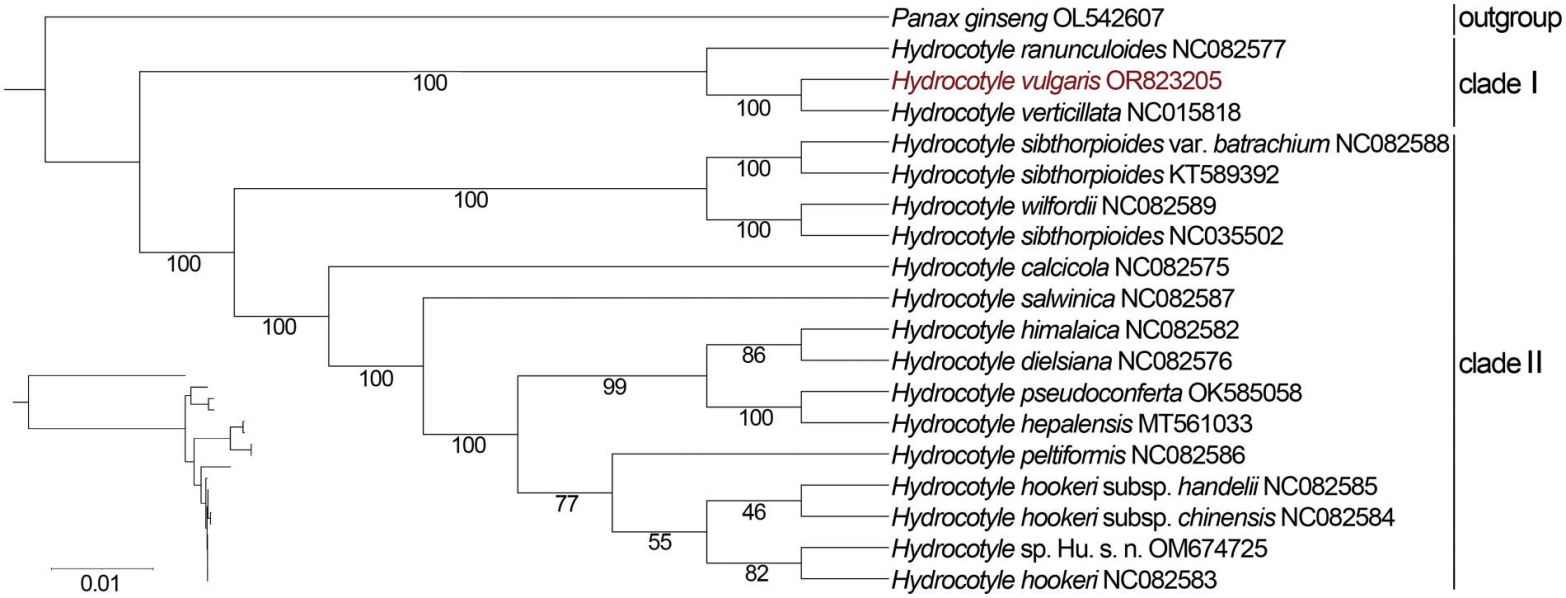
A maximum-likelihood phylogenetic tree based on the coding sequences of 78 common protein-coding genes from *Hydrocotyle* species. Bootstrap values next to the nodes are based on 1000 replications. *Panax ginseng* was set as the outgroup. GenBank accession numbers: *Panax ginseng* OL543607, *Hydrocotyle calcicola* NC082575, *Hydrocotyle dielsiana* NC082576, *Hydrocotyle himalaica* NC082582, *Hydrocotyle hookeri* subsp. *chinensis* NC082584, *Hydrocotyle hookeri* subsp. *handelii* NC082585, *Hydrocotyle hookeri* NC082583, *Hydrocotyle nepalensis* MT561038, *Hydrocotyle peltiformis* NC082586, *Hydrocotyle pseudoconferta* OK585058 (Wen et al. 2022), *Hydrocotyle ranunculoides* NC082577, *Hydrocotyle salwinica* NC082587, *Hydrocotyle sibthorpioides* NC035502, *Hydrocotyle sibthorpioides* var. *batrachium* NC082588, *Hydrocotyle sibthorpioides* KT589392 (Ge et al. 2017), *Hydrocotyle* sp. Hu s.n. OM674725, *Hydrocotyle verticillata* NC015818, and *Hydrocotyle wilfordii* NC082589.

The best model was VM + F + I. Based on 19 complete chloroplast genome sequences, a ML phylogenetic tree was constructed with *Panax ginseng* as an outgroup. As shown in Figure 3, the species of *Hydrocotyle* were divided into two clades with high bootstrap value. *H. ranunculoides* (NC082577), *H. vulgaris* (OR823205), and *H. verticillata* (NC015818) clustered in clade one, and other *Hydrocotyle* species into another clade. In addition, *H. vulgaris* has the closest relationship with *H. verticillata*.

## 5. Discussion and conclusions

Compared with the nuclear genome and mitogenome, the chloroplast genome of angiosperms is highly conserved, which provides a powerful tool for chloroplast gene function analysis, population genetic analysis, and phylogenetic evolutionary studies (Daniell et al. 2016; Mehmood et al. 2019). Additionally, the complete chloroplast genomes have been widely used in resolving some unanswered questions in plant taxonomy (Ruhsam et al. 2015). In this study, the complete chloroplast genome of *H. vulgaris* was obtained through high-throughput sequencing analysis, and we further analyzed its genomic structures, gene contents, SSRs, and the phylogenetic relationship among *Hydrocotyle* species. The phylogenetic tree showed that the species of *Hydrocotyle* clustered in one clade, which is consistent with the traditional taxonomy. This complete chloroplast genome sequence of *H. vulgaris* would provide a genetic resource for further genetic and genomic studies of the genus *Hydrocotyle*.

## Supplementary Material

Supplemental Material

Supplemental Material

Supplemental Material

## Data Availability

The genome sequence data that support the findings of this study are openly available in GenBank of NCBI at https://www.ncbi.nlm.nih.gov/ under the accession no. OR823205. The associated BioProject, SRA, and Bio-Sample numbers are PRJNA1041710, SRR26865521, and SAMN38289794, respectively.
