# Supplementary figures and images for: The complete chloroplast genome sequence of Hydrocotyle vulgaris L. (Araliaceae)
Source: Mitochondrial DNA B Resour. 2024 May 17;9(5):647–51. doi: 10.1080/23802359.2024.2349333 (PMC11104692; doi:10.1080/23802359.2024.2349333)

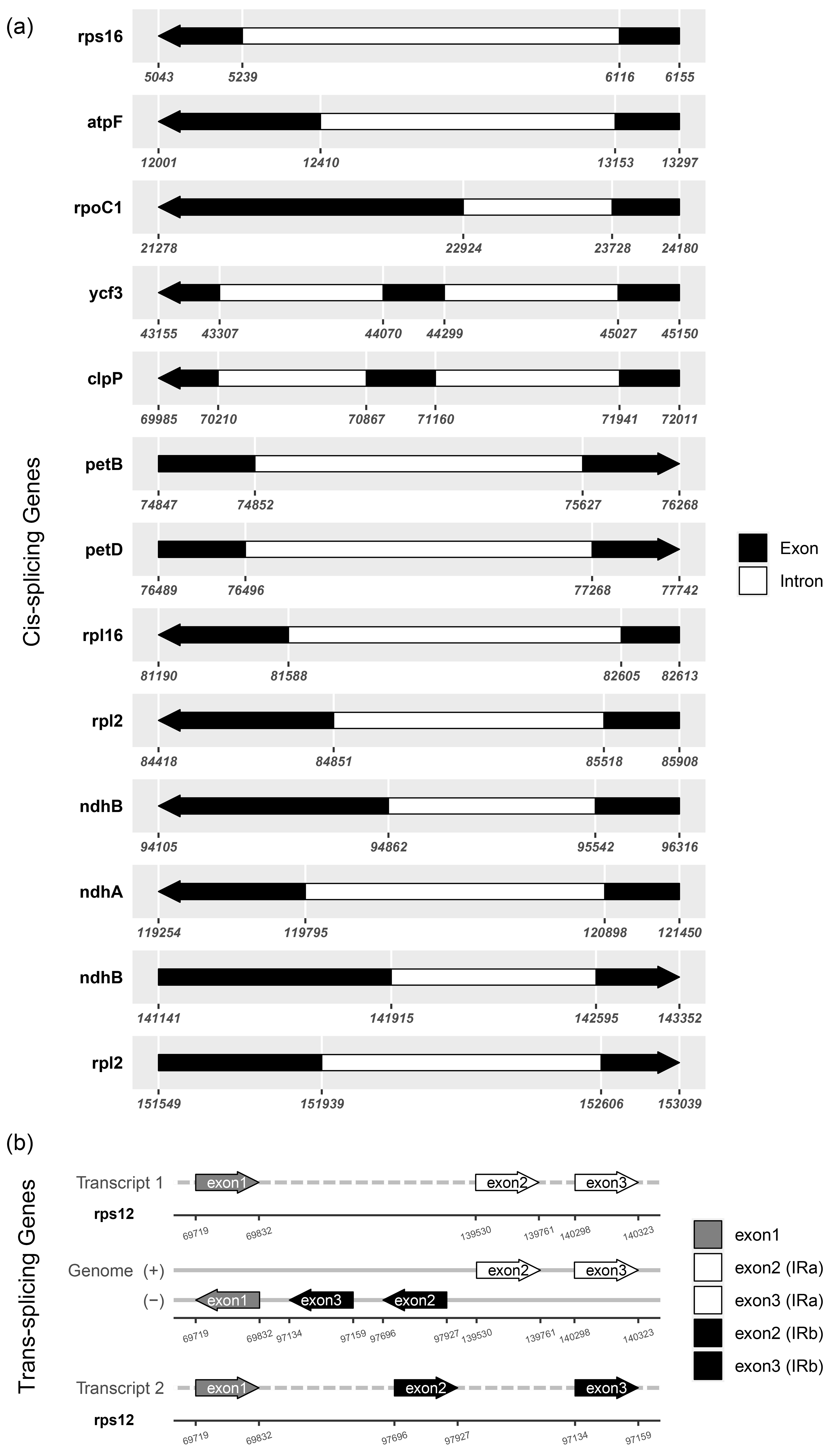

Supplement: Supplemental Material [file TMDN_A_2349333_SM5349.tif]

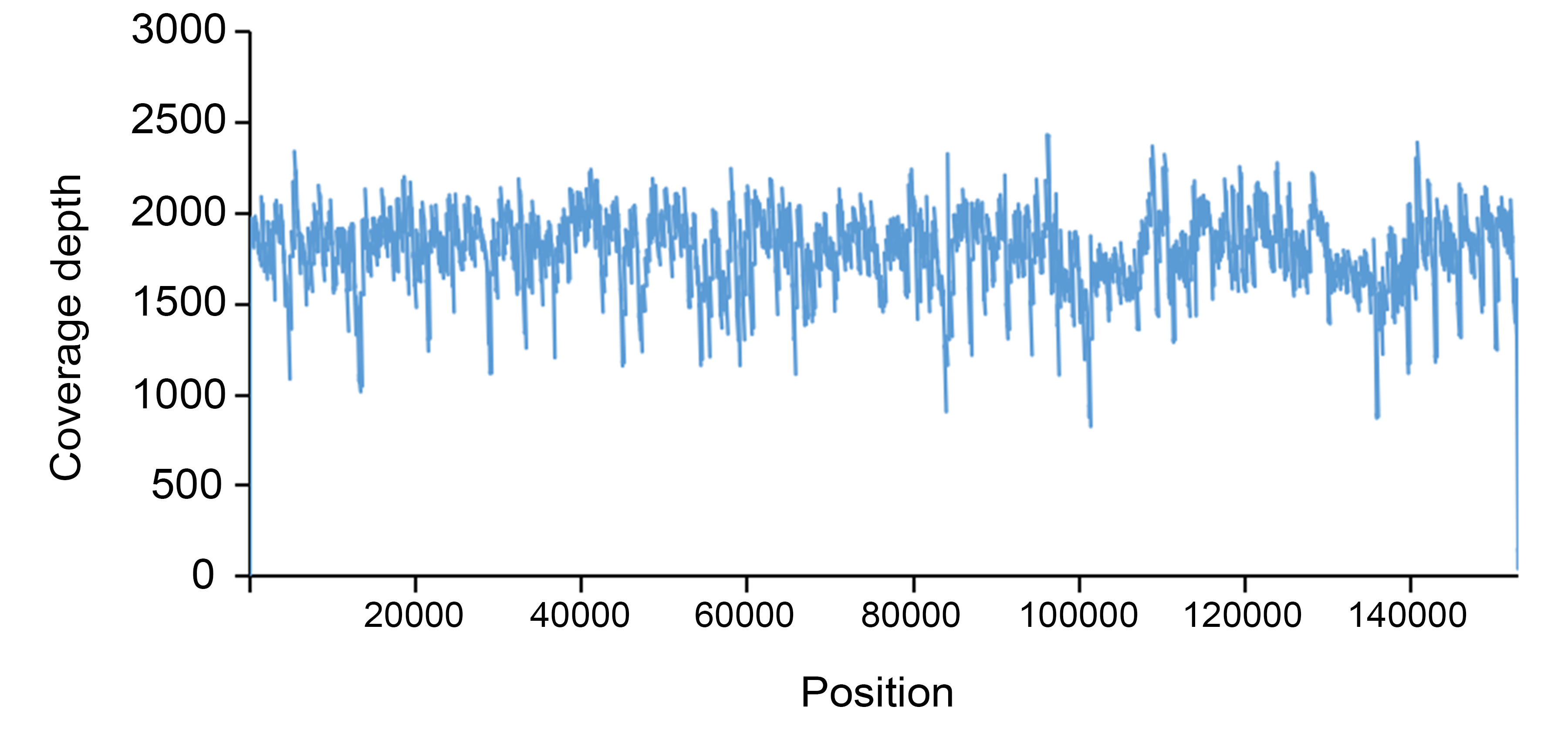

Supplement: Supplemental Material [file TMDN_A_2349333_SM5348.tif]
