# Supplementary material for: The complete chloroplast genome sequence of Hydrocotyle vulgaris L. (Araliaceae)
Source: Mitochondrial DNA B Resour. 2024 May 17;9(5):647–51. doi: 10.1080/23802359.2024.2349333 (PMC11104692; doi:10.1080/23802359.2024.2349333)
Supplement: Supplemental Material [file TMDN_A_2349333_SM5345.docx]

**Supplementary Table 1.** The lengths of introns and exons for the splitting genes in *H. vulgaris* chloroplast genome.

| **Gene Name** | **Strand** | **Start** | **End** | **ExonI** | **IntronI** | **ExonII** | **IntronII** | **ExonIII** |
| --- | --- | --- | --- | --- | --- | --- | --- | --- |
| *trnK-UUU* | - | 1606 | 4207 | 37 | 2530 | 35 |  |  |
| *rps16* | - | 5043 | 6155 | 40 | 876 | 197 |  |  |
| *trnG-UCC* | + | 9304 | 10074 | 23 | 700 | 48 |  |  |
| *atpF* | - | 12001 | 13297 | 145 | 742 | 410 |  |  |
| *rpoC1* | - | 21278 | 24180 | 453 | 803 | 1647 |  |  |
| *trnG-GCC* | + | 37019 | 37089 | 38 | 3 | 38 |  |  |
| *ycf3* | - | 43155 | 45150 | 124 | 727 | 230 | 762 | 153 |
| *trnL-UAA* | + | 48264 | 48863 | 37 | 514 | 49 |  |  |
| *trnV-UAC* | - | 51793 | 52444 | 39 | 578 | 35 |  |  |
| *clpP* | - | 69985 | 72011 | 71 | 780 | 294 | 656 | 226 |
| *petB* | + | 74847 | 76268 | 6 | 774 | 642 |  |  |
| *petD* | + | 76489 | 77742 | 8 | 771 | 475 |  |  |
| *rpl16* | - | 81190 | 82613 | 9 | 1016 | 399 |  |  |
| *rpl2* | - | 84418 | 85908 | 391 | 666 | 434 |  |  |
| *ndhB* | - | 94105 | 96316 | 775 | 679 | 758 |  |  |
| *trnI-GAU* | + | 101811 | 102826 | 37 | 944 | 35 |  |  |
| *trnA-UGC* | + | 102891 | 103767 | 38 | 804 | 35 |  |  |
| *ndhA* | - | 119254 | 121450 | 553 | 1102 | 542 |  |  |
| *trnA-UGC* | - | 133690 | 134566 | 38 | 804 | 35 |  |  |
| *trnI-GAU* | - | 134631 | 135646 | 37 | 944 | 35 |  |  |
| *ndhB* | + | 141141 | 143352 | 775 | 679 | 758 |  |  |
| *rpl2* | + | 151549 | 153039 | 391 | 666 | 434 |  |  |

**Supplementary Table 2.** Simple sequence repeats (SSRs) in *H. vulgaris* chloroplast genome.

| **Number** | **Type** | **SSR** | **Size** | **Start** | **End** | **Location** |
| --- | --- | --- | --- | --- | --- | --- |
| 1 | p1 | (A)11 | 11 | 2841 | 2851 | LSC |
| 2 | p1 | (A)17 | 17 | 4739 | 4755 | LSC |
| 3 | p2 | (AT)6 | 12 | 6888 | 6899 | LSC |
| 4 | p1 | (T)12 | 12 | 12689 | 12700 | LSC |
| 5 | p1 | (T)11 | 11 | 21412 | 21422 | LSC |
| 6 | p1 | (T)10 | 10 | 26753 | 26762 | LSC |
| 7 | p1 | (A)14 | 14 | 28708 | 28721 | LSC |
| 8 | p1 | (A)10 | 10 | 36098 | 36107 | LSC |
| 9 | p1 | (C)13 | 13 | 36768 | 36780 | LSC |
| 10 | p2 | (TA)6 | 12 | 44924 | 44935 | LSC |
| 11 | p1 | (T)12 | 12 | 45909 | 45920 | LSC |
| 12 | c | (TA)6gatatatatagaaaaagtattttttc(AT)6 | 50 | 47205 | 47254 | LSC |
| 13 | p1 | (T)11 | 11 | 51692 | 51702 | LSC |
| 14 | p1 | (T)10 | 10 | 54818 | 54827 | LSC |
| 15 | p1 | (A)14 | 14 | 55417 | 55430 | LSC |
| 16 | p1 | (A)10 | 10 | 66997 | 67006 | LSC |
| 17 | p1 | (T)13 | 13 | 70576 | 70588 | LSC |
| 18 | p1 | (T)11 | 11 | 78122 | 78132 | LSC |
| 19 | p1 | (T)11 | 11 | 80045 | 80055 | LSC |
| 20 | c | (T)10g(T)11 | 22 | 80560 | 80581 | LSC |
| 21 | p1 | (T)12 | 12 | 81068 | 81079 | LSC |
| 22 | p1 | (T)11 | 11 | 84009 | 84019 | LSC |
| 23 | p1 | (T)10 | 10 | 84375 | 84384 | IRa |
| 24 | p1 | (T)10 | 10 | 112712 | 112721 | SSC |
| 25 | p1 | (A)17 | 17 | 114240 | 114256 | SSC |
| 26 | p1 | (A)10 | 10 | 116688 | 116697 | SSC |
| 27 | p1 | (T)11 | 11 | 122734 | 122744 | SSC |
| 28 | p1 | (T)12 | 12 | 127132 | 127143 | SSC |
| 29 | p1 | (T)11 | 11 | 127407 | 127417 | SSC |
| 30 | p1 | (T)11 | 11 | 127583 | 127593 | SSC |
| 31 | p1 | (A)10 | 10 | 153073 | 153082 | IRb |

Note: Maximal number of bases interrupting 2 SSRs in a compound microsatellite.
